# Aberrant Expression of Pseudogene-Derived lncRNAs as an Alternative Mechanism of Cancer Gene Regulation in Lung Adenocarcinoma

**DOI:** 10.3389/fgene.2019.00138

**Published:** 2019-03-06

**Authors:** Greg L. Stewart, Katey S. S. Enfield, Adam P. Sage, Victor D. Martinez, Brenda C. Minatel, Michelle E. Pewarchuk, Erin A. Marshall, Wan L. Lam

**Affiliations:** ^1^BC Cancer Research Centre, Vancouver, BC, Canada; ^2^The Francis Crick Institute, London, United Kingdom

**Keywords:** long non-coding RNAs, pseudogenes, lung cancer, gene regulation, non-coding RNAs

## Abstract

Transcriptome sequencing has led to the widespread identification of long non-coding RNAs (lncRNAs). Subsequently, these genes have been shown to hold functional importance in human cellular biology, which can be exploited by tumors to drive the hallmarks of cancer. Due to the complex tertiary structure and unknown binding motifs of lncRNAs, there is a growing disparity between the number of lncRNAs identified and those that have been functionally characterized. As such, lncRNAs deregulated in cancer may represent critical components of cancer pathways that could serve as novel therapeutic intervention points. Pseudogenes are non-coding DNA sequences that are defunct relatives of their protein-coding parent genes but retain high sequence similarity. Interestingly, certain lncRNAs expressed from pseudogene loci have been shown to regulate the protein-coding parent genes of these pseudogenes in *trans* particularly because of this sequence complementarity. We hypothesize that this phenomenon occurs more broadly than previously realized, and that aberrant expression of lncRNAs overlapping pseudogene loci provides an alternative mechanism of cancer gene deregulation. Using RNA-sequencing data from two cohorts of lung adenocarcinoma, each paired with patient-matched non-malignant lung samples, we discovered 104 deregulated pseudogene-derived lncRNAs. Remarkably, many of these deregulated lncRNAs (i) were expressed from the loci of pseudogenes related to known cancer genes, (ii) had expression that significantly correlated with protein-coding parent gene expression, and (iii) had lncRNA protein-coding parent gene expression that was significantly associated with survival. Here, we uncover evidence to suggest the lncRNA-pseudogene-protein-coding gene axis as a prominent mechanism of cancer gene regulation in lung adenocarcinoma, and highlights the clinical utility of exploring the non-coding regions of the cancer transcriptome.

## Introduction

Lung cancer is an enormous health burden, representing the most common cause of cancer death worldwide. While advances in imaging technology have improved early detection, the outcome for lung cancer patients remains poor. This is largely attributed to limited therapeutic options and short-term response due to tumor heterogeneity. Despite extensive sequencing efforts to characterize exomes and protein-coding transcriptomes of lung tumors, less than half of lung adenocarcinoma (LUAD) patients harbor clinically targetable mutated driver genes, highlighting the need to explore alternative mechanisms of cancer gene deregulation (Chan and Hughes, 2015).

Long non-coding RNAs (lncRNAs, > 200 nt) have emerged as important players in cell biology. LncRNAs have been observed to function through a wide variety of regulatory mechanisms, targeting DNA, proteins, and other RNA species; specifically with defined roles in RNA degradation or stabilization, protein translocation and complex formation, and recruitment of complexes to transcriptional loci (Lee et al., 1999; Wang et al., 2011; Kung et al., 2013; Simon et al., 2013). This broad functional repertoire has been shown to be exploited by many cancer types, including LUAD, to drive various hallmarks of cancer (Bhan et al., 2017; Peng et al., 2017). One of the first cancer-associated lncRNA transcripts, *MALAT1* (Metastasis Associated Lung Adenocarcinoma Transcript 1) was discovered in lung cancer. *MALAT1* is overexpressed in metastatic lung tumors and functions *in trans* through transcriptional regulation of multiple genes involved in cell motility (Tano et al., 2010; Gutschner et al., 2013). Since the discovery of *MALAT1*, many other lncRNAs have been shown to play a direct role in nearly every major cancer type. Tumor suppressive lncRNA transcripts are commonly observed to be downregulated, for instance the lncRNA *TARID*, which promotes the expression of the tumor suppressor gene *TCF21* through active promoter demethylation (Arab et al., 2014). Conversely oncogenic lncRNAs can be overexpressed, such as *NEAT1*, an architectural lncRNA associated with metastasis in many cancer types (Hirose et al., 2014; Blume et al., 2015; Yamazaki et al., 2018; Zhang et al., 2018). As such, lncRNAs may represent critical regulators of oncogenic-driver pathways that could serve as undiscovered clinical intervention points in LUAD.

While lncRNAs have been observed to be important in cancer biology, functional prediction of newly-discovered lncRNAs remains a major challenge. However, many lncRNAs expressed from pseudogene loci have been shown to regulate the specific genes with which they have sequence homology. Pseudogenes are DNA sequences that are defunct relatives of functional protein-coding genes (herein referred to as parent genes) and arise during either gene duplication events, or the reverse transcription of an mRNA transcript into a new genomic location. Through evolution these duplicated genes have acquired mutations such as premature stop codons and frameshifts, which results in the loss of protein coding ability, while still retaining a high degree of sequence homology with the original parent gene (Khachane and Harrison, 2009). Recently, pseudogene-derived lncRNAs have been shown to regulate their parent genes and this novel mechanism has been observed in many tumor types, including lung cancer (Sun et al., 2017; Hu et al., 2018). A prominent example is the tumor suppressor gene *PTEN* (chromosome 10), regulated both positively by *PTENP1* (chromosome 9), a lncRNA transcribed from the sense strand of the pseudogene locus, and negatively by the lncRNA *PTENP1-AS1*, which is transcribed from the strand antisense to the parent gene(Johnsson et al., 2013).

Pseudogenes have been continually omitted from large RNA-sequencing datasets due to the complexity of separating highly similar pseudogene sequences from parent genes. However, Milligan et al. (2016) recently generated an atlas of lncRNAs overlapping pseudogenes, which has provided a foundation for their analysis in RNA sequencing datasets. We hypothesize that the functions of pseudogene-derived lncRNAs are an under-explored mechanism of gene regulation that occurs more broadly than previously realized, and that these events contribute to the tumorigenesis of LUAD. We performed next generation RNA-sequencing on microdissected LUAD tumors and matched non-malignant tissue to identify deregulated lncRNAs expressed from pseudogene loci (herein referred to as Ψ-lncs). We then explored the relationships of these Ψ-lncs with their parent genes, and explored their significance in relation to patient clinical features in our discovery dataset as well as a validation dataset.

## Results

### Ψ-lnc Expression Is Deregulated in Lung Adenocarcinoma

**FIGURE 1:**
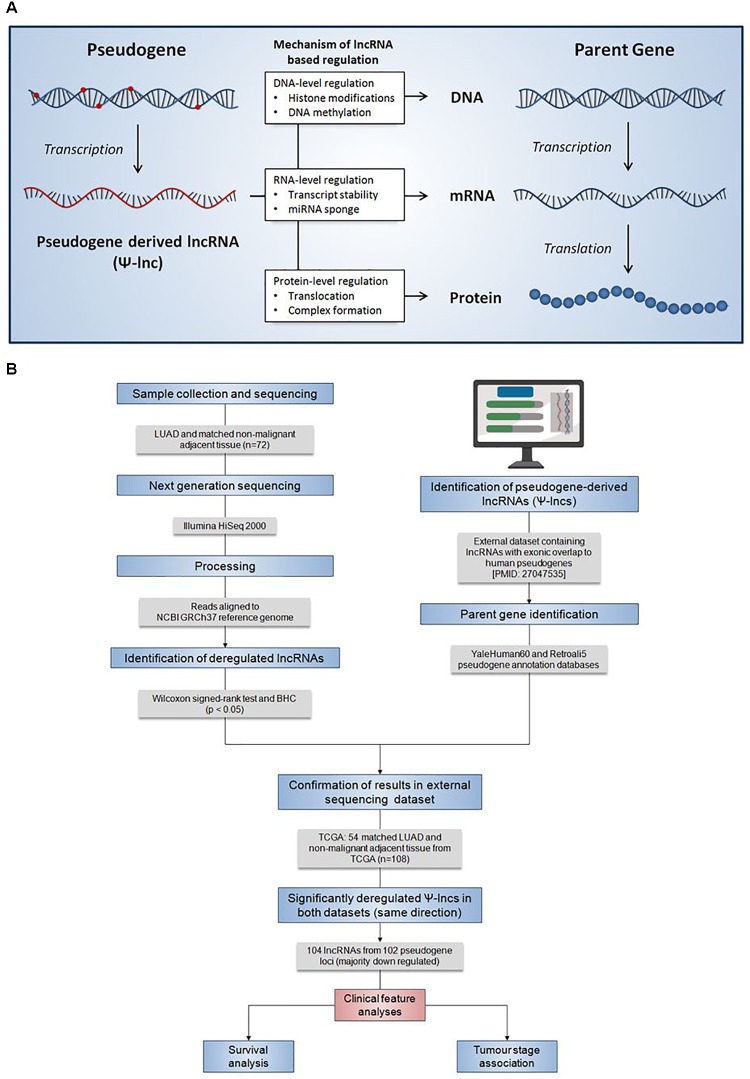
Summary of the regulatory mechanisms of Ψ-lncs and the analysis pipeline for the identification of their deregulation in lung adenocarcinoma. **(A)** Summary of the regulatory mechanisms of pseudogene-derived lncRNAs (Ψ-lncs) that retain sequence homology with the parent gene. Overall, lncRNAs have been shown to function through a variety of regulatory mechanisms, acting on the DNA, RNA, and protein levels. **(B)** Flow diagram description of the analysis pipeline applied for the identification of deregulated Ψ-lncs. Patient LUAD samples were collected and subjected to next generation sequencing to quantify RNA expression. Gene expression was then compared between tumors and matched non-malignant tissue to identify significantly deregulated transcripts. LncRNAs with exonic overlap to known pseudogenes were then identified and confirmed in a 2nd set of LUAD and matched non-malignant tissue. Deregulated Ψ-lncs were then assayed to determine associations with clinical features.

Pseudogenes vary widely in terms of length, gene fraction, and identity to parent genes, and can be expressed as lncRNAs that are sense, antisense, partial overlapping, or internal to the parent. In light of this variation, Ψ-lncs are observed to have vastly different regulatory effects on downstream target genes (Figure 1A). In our curation of Ψ-lncs in LUAD, we have included those that have exonic overlap with a pseudogene (partial or full length) and considered both sense and antisense transcripts (Supplementary Table S1). Ψ-lncs were analyzed in an in-house discovery (BCCA, *n* = 72) and external validation (TCGA, *n* = 108) cohort of LUAD and paired non-malignant lung tissues (Table 1). We identified aberrantly expressed Ψ-lncs that are significantly deregulated in both the discovery and validation datasets with the same direction of expression alteration (Ψ-lncs upregulated or downregulated in tumors compared to matched non-malignant tissue).

**Table 1:** Patient clinical characteristics of the discovery (BCCA), validation (TCGA), and survival analysis (KmPlotter) datasets.

Characteristic	BCCA	TCGA	KmPlotter
Samples (pairs)	72 (36)	108 (54)	866 (n/a)
Sex			
Male	10	24	344
Female	26	30	318
Average age	70	67	–
Stage			
I	20 (56%)	28 (52%)	370 (69%)
II	11 (31%)	14 (30%)	136 (25%)
III	3 (8%)	10 (19%)	24 (4%)
IV	1 (3%)	2 (4%)	4 (1%)
Ethnicity			
Caucasian	11 (31%)	51 (94%)	–
Asian	14 (39%)	–	–
Unknown	11 (31%)	–	–
Black	–	3 (6%)	–
Smoking			
Current	5 (14%)	7 (13%)	246 (Ever)^a^
Former	6 (17%)	36 (67%)	
Never	25 (69%)	5 (9%)	246

*^a^ Ever smokers is a term that includes both current and former smokers.*

**FIGURE 2:**
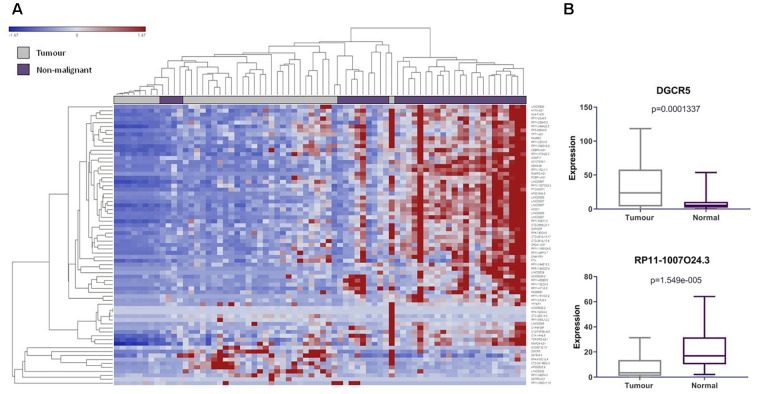
LncRNAs derived from pseudogene loci are significantly differentially expressed in lung adenocarcinoma compared to matched non-malignant lung tissue. **(A)** Unsupervised hierarchical clustering of pseudogene-derived lncRNAs differentially expressed between lung adenocarcinoma (gray) and matched non-malignant tissue (purple). Average linkage was used as the cluster distance metric and Pearson Correlation was used as the point correlation metric. Expression values are stratified from low (blue) to high (red). Only pseudogene-derived lncRNAs with average expression values of greater than or equal to 10 RPKM were included in the clustering analysis. Clustering of samples highlights relative similarity in pseudogene-derived lncRNA expression between the two sample groups, while clustering of gene expression reveals a trend toward the widespread underexpression of these transcripts in lung adenocarcinoma. **(B)** Highlighted examples of pseudogene-derived lncRNAs significantly deregulated between lung tumors and non-malignant tissues. Expression (RPKM) in tumors (gray) and normal tissues (purple) is represented on the Y-axis. Boxes represent the interquartile range and inner lines represent the median expression value.

We found 104 lncRNAs expressed from 102 pseudogene loci to be significantly deregulated in LUAD (Supplementary Table S2). To our surprise, we found that the majority of these deregulated Ψ-lncs were downregulated in tumors (Figure 2A,B). Most of these were unannotated lncRNAs, such as *RP11-1007O24.3*, which was downregulated in tumors, with only 24 of the total deregulated Ψ-lncs having been previously described in scientific literature annotated in PubMed, albeit none in the field of pseudogene-mediated deregulation (Supplementary Table S3 and Figure 2B). Twenty of these 24 have been described in the context of cancer, with only four in lung cancer. This includes *DGCR5*, a lncRNA we found to be overexpressed in tumors. *DGCR5* has been reported to promote LUAD progression by sequestering a variety of miRNAs involved in cell cycle regulation, although it has not been investigated with regard to its pseudogene-derived nature (Chen et al., 2017; Dong et al., 2018; Luo et al., 2018).

**FIGURE 3:**
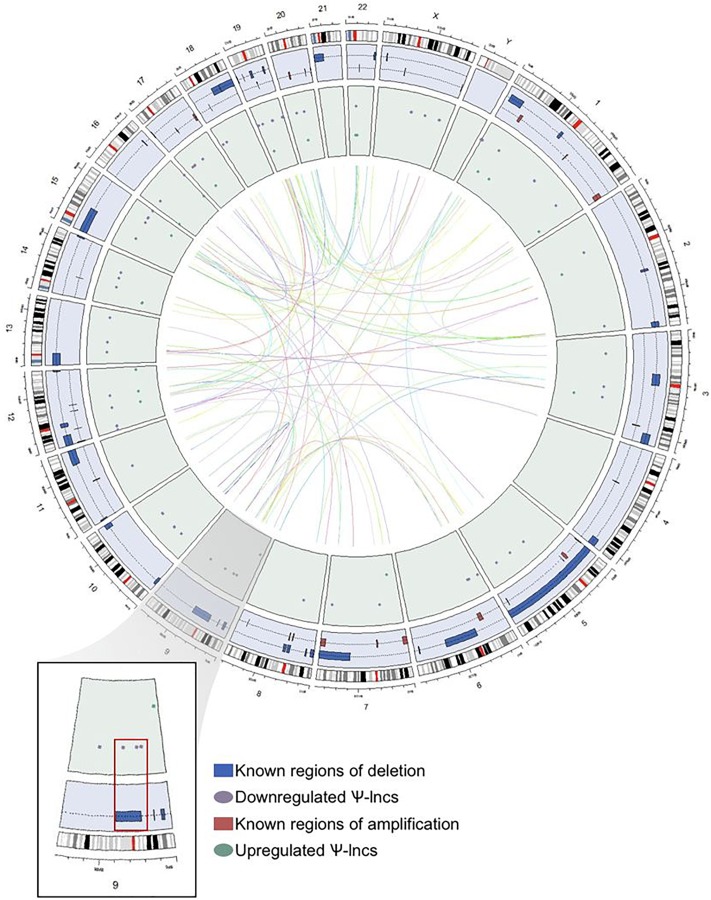
Genome-wide distribution of deregulated pseudogene-derived lncRNAs in lung adenocarcinoma. Circular representation of the genomic distribution of the deregulated pseudogene-derived lncRNAs discovered in our study, as well as known regions of copy number alterations in lung adenocarcinoma as described by TCGA. The outer concentric circle represents the human karyotype from the genomic build hg19. The blue concentric circle contains known regions of copy number amplification (red boxes) and deletion (blue boxes) that have been previously published. The inner green circle represents the specific genomic location of our pseudogene-derived lncRNAs found to be either upregulated (green circles) or downregulated (purple circles) in lung adenocarcinoma. Finally, the inner connecting lines represent the interaction between the deregulated pseudogene-derived lncRNAs and the locations of their respective protein-coding parent genes. Chromosome 9 (magnified region) highlights that some of the downregulated pseudogene-derived lncRNAs overlap with genomic regions frequently deleted in lung adenocarcinoma.

We were interested in examining the genetic events that could impact pseudogene loci, and thus affect Ψ-lnc expression. We mapped the chromosomal distribution of the deregulated Ψ-lncs, finding them to be distributed throughout the genome and detected on most chromosomes, except for chromosomes 4 and Y (Figure 3). The locations of each of the parent genes of deregulated Ψ-lncs are similarly distributed through the genome (Supplementary Table S4). We then determined the overlap of these genes with regions of recurrent chromosomal amplification and deletion as determined by The Cancer Genome Atlas (TCGA) for LUAD (Zack et al., 2013). While some Ψ-lncs overlap with regions of recurrent deletion, the majority do not, indicating that they may be regulated by mechanisms other than copy number alteration (Figure 3).

### Global Patterns of Ψ-lnc and Parental Gene Expression

As a first step to identify deregulated Ψ-lncs that may function through regulation of their respective parent genes, we explored whether Ψ-lncs with significantly deregulated expression were associated with altered parent gene transcript levels (Poliseno et al., 2010; Johnsson et al., 2013; Feng et al., 2017; Huang et al., 2018). We obtained parent gene information for the 95 deregulated Ψ-lncs and determined that they shared 104 parent genes. Some pseudogenes contained multiple lncRNAs, and some lncRNAs overlapped multiple pseudogenes, constituting a total number of 116 Ψ-lnc-parent gene pairings. For each Ψ-lnc-parent pair we compared groups of tumors with high levels of Ψ-lnc expression to those with low levels of Ψ-lnc expression.We found that 33 Ψ-lncs have a significant expression relationship with their parent gene in at least 1 dataset (Supplementary Table S5). This included 21 sense Ψ-lnc-parent-gene pairs, and 13 antisense Ψ-lnc-parent gene pairs. Having identified Ψ-lncs with expression associated with parent gene expression, we investigated whether parent genes had known oncogenic or tumor suppressive roles. We performed a literature search to determine if any had been previously described in the context of cancer. Interestingly, we found that 65 of these parent genes had been previously described in cancer, and of those, 33 had been described in lung cancer (Table 2). Of the 34 significantly differentially expressed Ψ-lnc-parent gene pairs, 25 parent genes were described in cancer. This includes lung cancer associated genes like *CS*, which affects tumor drug response, as well as *RCN1*, which is associated with poor prognosis and tumor progression in lung cancer (Chen et al., 2014, 2018).

**Table 2:** Parent genes of deregulated Ψ-lncs previously described in cancer literature.

Parent Gene	Cancer^a^	Lung^a^ Cancer	Ψ-lnc	Parent Gene	Cancer^a^	Lung^a^ Cancer	Ψ-lncRNA
*ADC*	8	–	*RP11-439L8.3*	*MT1*	>1000	95	*MMP24-AS1*
*AGAP1*	12	–	*AGAP11*	*NEBL*	17	–	*LINC00342*
*ARIH1*	4	–	*RP11-1007O24.3*	*NUP210*	9	2	*H1FX-AS1*
*ATXN7L3*	5	–	*RP11-56G10.2*	*PCBP2*	26	–	*PCBP1-AS1*
*BCR*	>1000	>1000	*AC008132.13*	*PKD1*	238	5	*RP11-1186N24.5*
*BMS1*	4	–	*AGAP11*	*POTEF*	4	–	*RP11-193H5.1*
*CDC42*	1657	129	*RP11-390F4.3*	*PPARGC1B*	37	1	*RP11-527N22.1*
*CECR7*	3	–	*AP000525.9;*	*PPY*	91	5	*CTD-2008P7.8*
			*CTD-2314B22.3*	*PRKX*	12	2	*RP11-526I2.1*
*CELSR1*	11	2	*DGCR5*	*PTCHD3*	2	–	*PTCHD3P1*
*CHRNB1*	8	–	*RP11-650L12.2*	*PTMA*	34	–	*LINC00987*
*CIC*	–	96	*RP11-34P13.7*	*PZP*	20	–	*LINC00987*
*CS*	–	10	*LINC00883;*	*RAB11FIP1*	16	1	*RP3-368A4.5*
			*RP11-446H18.5*	*RAB40B*	5	–	*LL0XNC01-250H12.3*
*CSPG4*	62	5	*DNM1P51*	*RCN1*	17	1	*TPT1-AS1*
*CTAGE1*	49	–	*RP1-122P22.2*	*RNASEH1*	7	–	*RP11-344E13.3*
*CYP4F2*	29	–	*CYP4F35P*	*RPL21*	5	–	*AC005062.2*
*CYP4F3*	10	1	*FAM95B1*	*RPL23A*	8	–	*HLA-F-AS1*
*CYP4F31P*	10	1	*CYP4F35P;*	*RPSA*	51	3	*FTX*
			*FAM95B1*	*RPSAP58*	–	3	*LINC00466*
*DFFB*	33	1	*TOPORS-AS1*	*SEMA3A*	163	25	*SEMA3B*
*DRD2*	121	10	*AP000438.2*	*SHQ1*	10	1	*RP11-50E11.3*
*EGLN1*	158	5	*RP11-182J1.1*	*SNAPC5*	4	–	*LY86-AS1*
*FAM103A1*	1	–	*RP11-324H6.5*	*SNX18*	2	–	*RP11-435B5.5*
*GPR39*	8	–	*RP11-399O19.9*	*SRSF9*	5	–	*RP11-752G15.3*
*HMGB1*	>1000	111	*RP11-349A22.5;*	*TACC3*	122	21	*LINC00667*
			*ZBED3-AS1*	*TOMM40*	10	2	*CTD-2314B22.3*
*HMGN2P46*	81	1	*RAMP2-AS1*	*TPTE*	69	7	*TPTEP1*
*KRT8*	94	7	*RP5-1198O20.4*	*TUBB4B*	5	–	*RP11-386G11.10*
*LINC00657*	2	–	*CTA-14H9.5;*	*TULP3*	5	–	*LINC00359*
			*HCG11*	*VENTX*	8	1	*RP11-81H3.2*
*LMNB2*	3	1	*RP11-161H23.9*	*VWF*	733	52	*TPTEP1*
*MARK4*	25	3	*CTD-2201G3.1*	*ZNF14*	2	–	*CTD-2666L21.1;*
*MICE*	1	–	*HLA-F-AS1*				*ZNF833P*
*MIPEPP3*	4	1	*C1QTNF9B-AS1*	*ZNF44*	1	–	*CTD-2666L21.1*
				*ZNF584*	1	–	*CTD-2619J13.17*

*^a^ denotes number of entries in PubMed.*

**FIGURE 4:**
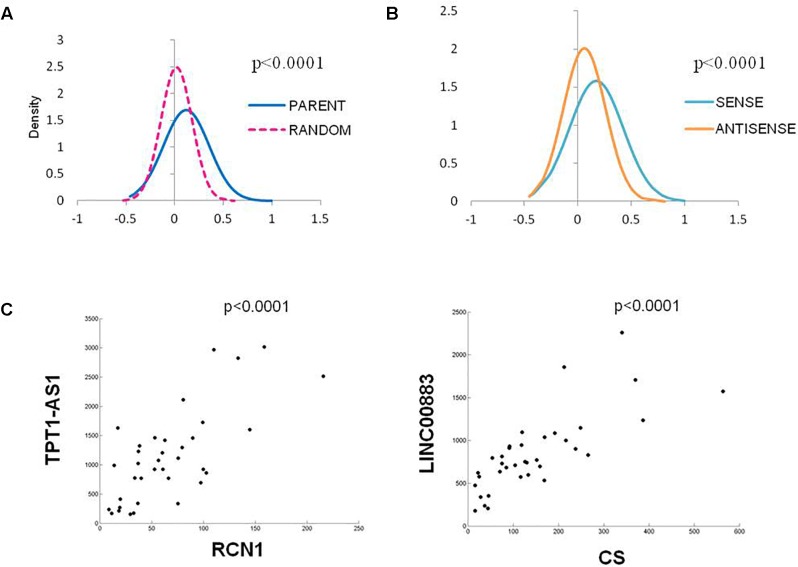
Distribution of Spearman’s Correlation rho values for all Ψ-lnc-parent-gene pairs in the TCGA dataset (*n* = 391). **(A)** Distribution of Spearman’s correlation coefficients between Ψ-lnc-parent gene pairs (blue line, median *R* = 0.088) and Ψ-lnc-random gene pairs (pink dashed line, median *R* = 0.019). Rho values of the groups were compared by Mann Whitney *U*-test. **(B)** Distribution of Spearman’s correlation coefficients between sense Ψ-lnc-parent-gene pairs (turquoise line, median *R* = 0.140) and antisense Ψ-lnc-parent-gene pairs (orange line, median *R* = 0.049). **(C)** Correlation scatter plots of Ψ-lncs with positive expression correlations to cancer-associated parent genes.

As the vast majority of the deregulated Ψ-lncs that were correlated with their parent gene had positive associations, we were interested whether this was a global phenomenon or exclusive to deregulated genes. We performed a Spearman’s correlation analysis on every Ψ-lnc-parent-gene pair with expression data in our dataset irrespective of deregulation status (*n* = 390 gene pairs). We plotted the distribution of Spearman’s rho (ρ) values for the Ψ-lnc-parent-gene pairs and compared them to the rho values for Ψ-lncs paired to randomly selected genes. We found that the Ψ-lnc-parent-gene pairs have significantly more positive relationships than the random gene pairs in both the BCCA (Mann Whitney *U*-test, *p* < 0.0001) and TCGA datasets (Mann Whitney *U*-test, *p* < 0.0001; Figure 4A). Studies have shown that lncRNAs transcribed from opposite strands can have different regulatory effects on target genes (Johnsson et al., 2013; Balbin et al., 2015). To determine if transcriptional orientation has an effect on Ψ-lnc-parent relationships we compared the Spearman’s rho values of sense Ψ-lnc-parent pairs to antisense Ψ-lnc-parent pairs. In both datasets we observed that the sense Ψ-lnc-parent pairs to have significantly more positive relationships than the antisense Ψ-lnc-parent pairs (Mann Whitney *U*-test, TCGA set (*p* < 0.0001), and BCCA set (*p* < 0.0025) (Figure 4B and Supplementary Table S6). Strongly positively correlated Ψ-lnc-parent pairs include *TPT1-AS1*/*RCN1* and *LINC00887*/*CS* (Figure 4C).

### Ψ-lncs and Their Parent Genes Are Associated With Patient Survival

If the aberrant expression of Ψ-lncs is biologically relevant, it follows that they may be relevant in tumor aggressiveness, stage, and patient survival. We performed a two-group analysis using a Mann Whitney *U*-test between Stage I tumors and Stage II-IV tumors, as the majority of our tumors fell into these categories (Table 1). Of the deregulated Ψ-lncs we found *CTC-250I14.3* to be associated with Stage 1 disease and downregulated in both the BCCA and TCGA LUAD cohorts (Supplementary Figure S1). Our discovery datasets were limited in sample size for survival association analysis; therefore, we examined a third cohort of 719 LUAD from the KM Plotter database (Kaplan–Meier Plotter)^1^ (Table 1). This cohort was limited to genes with probe coverage on microarray platforms. A total of 19 of the deregulated Ψ-lncs were represented on this platform, yet, the majority of these Ψ-lncs (16 of 19) were significantly associated with poor overall survival (log-rank *p* < 0.05, Table 3).

**Table 3:** Associations between Ψ-lncRNA, parent gene expression, and patient outcome.

Ψ-lncRNA	Survival *p* (KmPlotter)	Parent Gene	Survival *p* (KmPlotter)	Association with Parent (*p*, BCCA)	Association with Parent (*p*, TCGA)
*AC008132.13*	–	*BCR*	0.00043^a^	0.0173	–
*AGAP11*	0.0099^b^	*AGAP1*	5.80E-10^b^	0.0005	–
*AGAP11*	–	*BMS1*	0.00018^a^	0.0121	–
*AP000525.9*	–	*CECR7*	3.20E-06^a^	–	0.0371
*CTA-14H9.5*	–	*LINC00657*	6.00E-06^b^	–	–
*CTD-2314B22.3*	–	*CECR7*	3.20E-06^a^	–	0.0402
*CTD-2619J13.17*	–	*ZNF584*	1.70E-08^a^	–	0.0205
*CTD-2666L21.1*	–	*ZNF44*	2.00E-15^b^	–	0.0013
*CTD-2666L21.1*	–	*ZNF14*	1.90E-12^a^	–	–
*DGCR5*	4.00E-04^a^	*CELSR1*	2.70E-02^a^	–	–
*FAM66C*	3.00E-04^b^	*DEFB130*	–	–	–
*FAM95B1*	–	*CYP4F3*	2.00E-01^b^	–	–
*HCG11*	1.40E-08^b^	*LINC00657*	6.00E-06^b^	–	0.0046
*LINC00342*	1.80E-06^b^	*NEBL*	2.30E-05^b^	–	–
*LINC00466*	4.60E-06^a^	*RPSAP58*	–	–	–
*LINC00639*	0.0087^b^	*ZFP41*	0.014^b^	–	–
*LINC00667*	4.50E-08^b^	*TACC3*	6.20E-09^a^	–	–
*LINC00883*	2.90E-03^b^	*CS*	0.00041^a^	0.0449	–
*LINC00957*	0.042^b^	*RASA4B*	0.035^a^	–	–
*LINC00982*	1.30E-06^b^	*n/a*^c^	–	–	–
*LINC00987*	–	*PZP*	0.02^a^	–	<0.0001
*LY86-AS1*	0.00025^b^	*SNAPC5*	5e-0.6^b^	–	–
*MMP24-AS1*	0.023^a^	*MT1*	1.20E-12^a^	–	–
*PCBP1-AS1*	–	*PCBP2*	4.30E-13^b^	–	–
*RAMP2-AS1*	0.014^b^	*HMGN2P46*	–	–	–
*RP11-1007O24.3*	–	*ARIH1*	0.00093^b^	<0.0001	0.0030
*RP11-1186N24.5*	–	*PKD1*	0.0013^b^	–	<0.0001
*RP11-182J1.1*	–	*EGLN1*	1.20E-08^b^	0.0011	–
*RP11-344E13.3*	–	*RNASEH1*	1.00E-07^b^	0.0007	–
*RP11-349A22.5*	–	*HMGB1*	7.50E-10	–	–
*RP11-34P13.7*	–	*CIC*	1.20E-08^a^	–	0.0205
*RP11-390F4.3*	–	*CDC42*	1.30E-05	0.0449	–
*RP11-439L8.3*	–	*ADC*	3.30E-09	0.0205	–
*RP11-446H18.5*	–	*CS*	0.00041^a^	–	–
*RP11-93K22.13*	–	*FAM86B3P*	6.30E-05^b^	–	0.0129
*TOPORS-AS1*	–	*DFFB*	9.10E-08^b^	–	0.0033
*TPT1-AS1*	0.00019^b^	*RCN1*	0.016^a^	0.0242	–
*ZBED3-AS1*	1.10E-10^b^	*HMGB1*	7.50E-10^b^	0.0100	–
*ZNF833P*	–	*ZNF491*	0.016^b^	–	–
*ZNF833P*	–	*ZNF14*	1.90E-12^a^	–	0.0079

*^a^ Denotes poor overall survival associated with high gene expression. ^b^ Denotes poor overall survival associated with low gene expression. ^c^ No information for the parent gene.*

**FIGURE 5:**
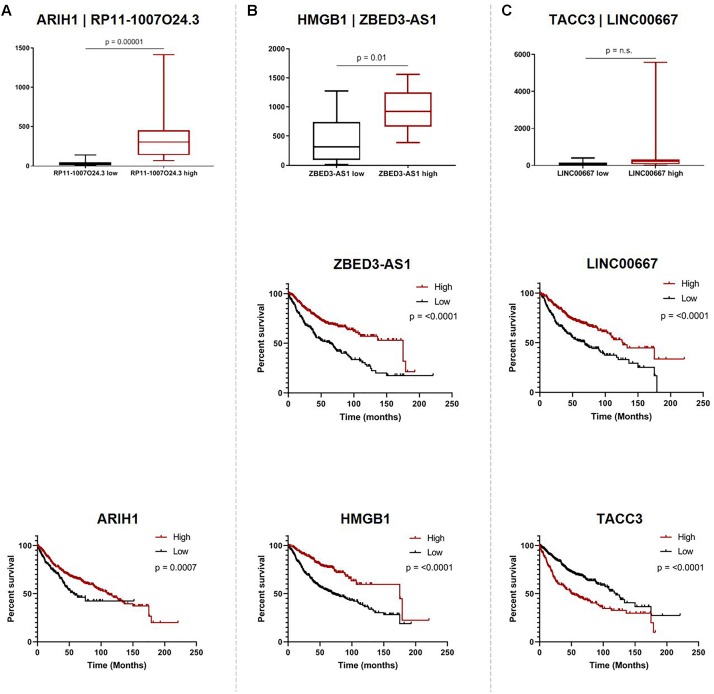
Associations of pseudogene-derived lncRNAs (upper row), their respective parent gene expression levels and their potential impact on patient outcome (middle row and bottom row). Expression and survival associations as seen for: **(A)** *RP11-1007O24.3* and parent gene *ARIH1*, **(B)** *ZBED3-AS1* and parent gene *HMGB1*, and **(C)** *LINC00667* and parent gene *TACC3*. Expression associations (upper row) for each pseudogene-derived lncRNA and parent gene pair were found by stratifying samples into tertiles by high (red) and low (black) expression of the pseudogene-derived lncRNA, and plotting the expression of the parent-gene (RPKM) on the y-axis. Survival associations were found for pseudogene-derived lncRNAs (middle row) and their respective parent genes (bottom row). Samples were stratified into tertiles with high (red) and low (black) expression of the gene-of-interest, and the significance of the associations were assessed using the logRank method through GraphPad Prism 8 software on data obtained from kmPlot (*n* = 673). Survival information was not available for RP11-1007O24.3 and it was thus omitted from this analysis.

While we were not able to investigate the survival associations of all deregulated Ψ-lncs as many were not covered by the microarray platforms, the majority (72 out of 103) of their parent genes were represented. We discovered that 67 of these parent genes were associated with patient survival. Twenty-eight of these survival associated parent genes were also significantly associated with the expression of their paired deregulated Ψ-lnc. Furthermore, we found 11 pairs where both Ψ-lnc and parent gene are associated with patient survival. For example *RP11-1007O24.3*, a Ψ-lnc downregulated in tumors, is positively associated with expression of survival-associated parent gene *ARIH1* in both the BCCA and TCGA datasets (Table 3 and Figure 5A). Further examples also include Ψ-lnc-parent pairs such as *ZBED3-AS1* and *HMGB1*, which are positively correlated at the expression level, and both significantly associated with survival (Figure 5B). We also observe Ψ-lnc-parent pairs that are associated with survival, but do not share an expression relationship such as *LINC00667* and parent gene *TACC3* (Figure 5C). Collectively, our discovery of the broad deregulation of Ψ-lncs, many of which are survival-associated and associated with parent gene expression, may indicate that Ψ-lncs impact LUAD biology through *trans* regulation of their cancer-associated parent genes.

## Discussion

Here we expand upon the work done by Milligan et al. (2016), completing the first large-scale analysis of lncRNA expression from pseudogene loci in LUAD and paired non-malignant lung tissue. We discovered a broadly positive association between Ψ-lncs and parent-gene expression, suggestive of an alternative mechanism of cancer gene regulation. While there have been singular examples of deregulated pseudogene-derived lncRNAs in cancer, we show that this phenomenon is widespread in LUAD. In addition to being correlated with Ψ-lnc expression, we find that many of the parent genes of these deregulated lncRNAs are annotated cancer genes and are significantly associated with patient survival, highlighting how these previously unappreciated non-coding genes may affect LUAD biology.

While the identification of lncRNAs associated with cancer phenotypes is increasing, a great challenge in the field remains the accurate downstream prediction of lncRNA function. Unlike protein-coding genes or small non-coding RNAs, features like complex folding patterns and unknown binding motifs have contributed to the challenging functional characterization of lncRNAs. We utilized the sequence similarity found between lncRNAs expressed from pseudogene loci, and their parent genes to predict the function of this subset of lncRNAs in LUAD. We identified a set of 104 Ψ-lncs deregulated in LUAD in two independent datasets. This greatly increases the number of deregulated lncRNAs known to be expressed from pseudogene loci in LUAD. Interestingly, the majority of these Ψ-lncs were under-expressed in tumors compared to non-malignant tissues, suggesting that they may have tumor-suppressive roles, and that their downregulation is advantageous to LUAD tumorigenesis.

Under-expression was not significantly associated with regions of recurrent copy number deletion in LUAD, although a subset of deregulated Ψ-lnc loci were localized to these regions (Figure 3). These observations suggest that they may be regulated by alternative molecular mechanisms, including broad chromosomal aberrations that affect whole chromosome arms, or epigenetic mechanisms. For example, endogenous retroviruses and repetitive elements often become aberrantly expressed in cancer due to deregulated methylation patterns (Kassiotis, 2014). We did not observe enrichment of Ψ-lncs or their parent genes on any chromosomes, despite the fact that pseudogenes are known to be overabundant on the human X chromosome (Drouin, 2006).

The direction of transcription often affects lncRNA function (Balbin et al., 2015). For example, the *PTEN* pseudogene (*PTENP1*) expressed in the sense direction can function as a decoy for inhibitory miRNAs that would otherwise cause translational inhibition of the *PTEN* parent mRNA. Conversely, when the antisense lncRNA is expressed from the *PTENP1* locus, the transcript is able to localize to the *PTEN* parent locus and recruit chromatin-remodeling machinery, which leads to the silencing of *PTEN* transcription (Poliseno et al., 2010; Johnsson et al., 2013). Both mechanisms have been coopted by cancer cells for their respective tumor suppressive and oncogenic roles (Hu et al., 2018). We found sense Ψ-lnc-parent pairs, (which account for 208 out of 391 Ψ-lncs examined) to be more positively correlated than antisense Ψ-lnc-parent pairs in both cohorts (Figure 4B). This may imply that Ψ-lncs are more likely to regulate their parent gene in a positive manner which may occur through mechanisms such as miRNA sponging or transcript stabilization when transcribed in the sense direction (Glenfield and McLysaght, 2018; Hu et al., 2018). The distribution of Spearman’s ρ-values for antisense-parent gene pairs suggests a more even split between positive and negative regulation. A limitation of this study is that we cannot discount the possibility that sequencing reads for sense overlapping Ψ-lncs that have sequence homology with their parent gene are being mapped to the parent gene instead of the Ψ-lnc. This potential issue warrants further investigation considering both the large number of annotated pseudogenes in the genome (*n* = 13,000) and the possibility of false interpretation of sequencing data for both protein-coding and non-coding genes.^2^ While these alignment errors could affect sense Ψ-lnc-parent gene pairs, antisense Ψ-lnc-parent gene pairs are not subjected to this same technical artifact. Recently, RNA-sequencing analysis strategies have emerged that begin to address this issue, and long read RNA-sequencing could be used to reduce errors in sequence alignment.

When looking at the parent genes of these deregulated Ψ-lncs we were interested to find that many had previously described roles in cancer (Table 2). This includes *EGLN1*, a well described cancer gene involved in regulation of tumor hypoxia, and *CDC42*, an oncogene involved in cell cycle control (Chan and Giaccia, 2010; Maldonado and Dharmawardhane, 2018). Many of these deregulated Ψ-lncs were also associated with clinical parameters such as patient survival and patient stage, in addition to the correlated expression between Ψ-lncs and their parent genes (Table 3). For example, *ZBED3-AS1* and *HMGB1* were positively correlated at the expression level, and low expression of both genes was associated with poor patient survival (Figure 5B). We also observed Ψ-lnc-parent pairs where both genes are associated with survival, but do not share an expression relationship. *LINC00667* and parent gene *TACC3*, for example, are both survival-associated, but not correlated at the expression level. *TACC3* is a component of the TACC3/ch-TOG/clathrin protein complex, and roles in complex assembly have been previously observed for lncRNAs (Munschauer et al., 2018; Figure 5C). Thus, it is possible that *LINC00667* is involved in a form of regulation that would not affect transcript levels, including protein-complex assembly. While we were unable to assess survival associations for many of the deregulated Ψ-lncs, they may still impact patient survival through regulation of their parent genes. We found 28 out of 34 expression-associated Ψ-lnc-parent gene pairs to have parent genes associated with patient survival. *RP11-1007O24.3*, for example, was positively correlated with survival-associated parent gene *ARIH1* expression in both cohorts. ARIH1 has been previously described to be a mediator of DNA-damage response and mitophagy in cancer cells (Figure 5A; Perdomo et al., 1988; von Stechow et al., 2015). The potential regulatory impact of Ψ-lncs on their clinically relevant parent genes is considerable and may represent a novel avenue for targeted therapies. While this study focused on the broad effects of Ψ-lnc deregulation, future studies utilizing *in vitro* and *in vivo* experiments will be necessary to determine the specific mechanisms of parent gene regulation.

As Ψ-lncs may represent an unexplored area of cancer-associated parent gene regulation, their therapeutic relevance should be further explored. LncRNAs make ideal targets for therapies that target RNA products such as Antisense Oligonucleotide (ASO) therapies, since RNA is their final functional state, rather than the intermediate product for protein-coding genes (Crooke et al., 2018). In addition, ASOs are easier and less costly to develop than small molecule inhibitors, and are in development as aerosol sprays that may be ideal for lung cancer treatment (Kjems and Howard, 2012; Li and Chen, 2013). However, as ASOs target through complementary sequence pairing, they would have to be designed in such a way as to not interfere with the parent gene, especially in the case of Ψ-lncs expressed from the sense strand.

This strategy of identifying lncRNAs aberrantly expressed from pseudogene loci may be useful when applied to other cancer types. Indeed, we see that several of our deregulated Ψ-lncs have been described in other tumor types, such as *TPT1-AS1* in cervical cancer, and *HGC11* in both prostate cancer and hepatocellular carcinoma (Zhang et al., 2016; Xu et al., 2017; Jiang et al., 2018). Additionally, as lncRNA expression is highly tissue specific, the application of this approach to other cancer types may yield novel disease-specific Ψ-lnc-parent pairs, highlighting the clinical utility of examining these previously underappreciated transcripts. Overall, Ψ-lnc-cancer-parent-gene axes represent alternative mechanisms of cancer gene regulation, and their identification is a critical step toward the functional characterization of lncRNAs.

## Conclusion

There is a growing need to functionally characterize lncRNAs. Pseudogene-derived lncRNAs have been shown to be involved in cancer and regulate the expression of their parent genes. We show here how pervasive this gene regulatory mechanism is in LUAD samples. We identify a large set of deregulated Ψ-lncs, with aberrant expression observed in RNA-sequencing data from two LUAD cohorts of paired tumor and non-malignant lung tissue samples. We show that these deregulated Ψ-lncs have clinical value and that the parent genes, many of which are correlated with Ψ-lnc expression, have been implicated in cancer phenotypes and are associated with clinical outcome Together, our results highlight the important roles of the non-coding transcriptome in cancer cellular biology.

## Materials and Methods

### Next Generation Sequencing of Lung Adenocarcinoma Patient Samples

#### Discovery Cohort

We performed Next Generation Illumina HiSeq RNA sequencing on a set of 36 micro-dissected LUAD tumors and matched adjacent non-malignant tissue (*n* = 72). Our British Columbia Cancer Agency cohort (BCCA) was composed of fresh-frozen LUAD tumors and matched non-malignant lung parenchymal tissue collected from 36 patients at the Vancouver General Hospital with approval from the University of British Columbia-BCCA Research Ethics Board. Consent obtained from the tissue donors of this study was both informed and written. To avoid field effects non-malignant samples were collected from areas > 2 cm away from the tumor. In order to reduce contaminating sequences derived from alternative cell types, tissue microdissection was guided by a pathologist. Samples used in this study contained > 80% tumor cell or > 80% non-malignant cell content. Total RNA was extracted using Trizol reagent and standard procedures.

#### Processing of RNA-Sequencing Data

Total RNA was used for library construction at the Genome Sciences Center (GSC, Vancouver, BC, Canada). Briefly, samples were first analyzed using Agilent Bioanalyzer RNA nanochip, and samples that passed quality check were arrayed into a 96-well plate. PolyA+ RNA was purified using the 96-well MultiMACS mRNA isolation kit on the MultiMACS 96 separator (Miltenyi Biotec, Germany) from 2 μg total RNA with on-column DNase I-treatment as per the manufacturer’s instructions. Double-stranded cDNA was synthesized from the purified polyA^+^-RNA using the Superscript Double-Stranded cDNA Synthesis kit (Life Technologies, United States) and random hexamer primers at a concentration of 5 μM. The paired-end sequencing library was prepared following the GSC paired-end library preparation protocol, which is strand specific. Sequencing was performed using the Illumina HiSeq 2000 platform. Raw sequencing reads were subject to a quality control process. Reads with a length < 50 nt (under two thirds of maximum read length of 75 nt) and quality level (Phred) < 20 were discarded. High quality reads (.fastq files) were aligned to the NCBI GRCh37 reference human genome build using the STAR aligner (v 2.4.1d) under default parameters (Dobin et al., 2013). Aligned reads (.bam files) were quantified using Ensembl Transcripts (Release 75) reference annotations (Flicek et al., 2014). Raw RNA sequencing reads from each patient (tumor and corresponding non-malignant tissue) were deposited at BioProject.^3^ Quantification was performed using the Partek Flow platform as reads per kilobase per million (RPKM). RNA sequencing (.bam files) and clinical data for a secondary set of LUAD tumors and matched non-malignant tissue (*n* = 108) were downloaded from The Cancer Genome Atlas (TCGA) Data Portal for validation purposes^4^ (Table 1). Expression profiles from TCGA were processed as described above.

### Identification of lncRNAs Expressed From Pseudogene Loci and Corresponding Parent Genes

#### Ψ-lnc Annotation

Milligan et al. (2016) recently published a global atlas of lncRNAs that have exonic overlap with positionally non-redundant (unique) pseudogenes from 3 major pseudogene databases. Using this resource we obtained a list of lncRNAs overlapping pseudogene loci (Supplementary Table S1) that we used as a foundation for our expression analysis. As the degree of sequence overlap required for a pseudogene-derived lncRNA to regulate its parent gene is unknown, we did not restrict our analysis to full-length, expressed pseudogenes, and included lncRNAs with any exonic overlap, including sense and antisense transcripts in order to annotate the most comprehensive list of Ψ-lncs (lncRNAs overlapping pseudogene loci).

#### Parent Gene Annotation

The parent gene information was also extracted for all pseudogenes overlapping our list of Ψ-lncs that have parent-gene annotations in the YaleHuman60 and Retroali5 databases (Supplementary Table S7). Manual literature search was performed for parent genes of deregulated Ψ-lncs that were not contained in these databases.

### Statistical Analysis

#### Identification of Significantly Deregulated Ψ-lncs in Paired LUAD and Non-Malignant Lung Tissue

Gene expression for protein-coding and non-coding genes was compared between tumors and non-malignant tissue and significantly deregulated genes were identified using a Wilcoxon signed-rank test (*p* < 0.05) and subjected to a Benjamini-Hochberg (BH) FDR correction. Ψ-lncs as identified previously were extracted, and those that were significantly deregulated between tumors and non-malignant tissue, in both our discovery (BCCA) and validation (TCGA) were selected for further analysis (Figure 1B).

#### Ψ-lncs and Parent Gene Expression

Tumors were sorted by Ψ-lnc expression for each Ψ-lnc-parent gene pair, and grouped into top and bottom Ψ-lnc expressing tertiles. Parent gene expression was then compared between the two groups using the Mann Whitney *U*-test (*p*-value ≤ 0.05). We performed a global expression analysis to determine whether Ψ-lnc-parent pairs were more positive or negatively correlated than random chance. For all Ψ-lncs with expression data in both datasets, Spearman’s correlation rho values were calculated for Ψ-lnc-parent gene pairs (*n* = 390) and compared to Ψ-lnc-random gene pairs. Random genes in our expression matrices were selected to pair with each Ψ-lnc. Each gene was assigned a number and pairs were chosen by using a random number generator.^5^ Spearman’s rho values were then plotted, smoothed, and compared (Mann Whitney *U*-test, *p*-value ≤ 0.05). Rho value distribution was also compared between sense lnc-parent gene pairs (*n* = 208) and antisense lnc-parent pairs (*n* = 182).

#### Literature Searches

To determine if each gene of interest (Ψ-lnc or parent gene) had been previously described in the context of tumors we searched Pubmed using the terms “gene + cancer” or “gene + lung cancer.”

#### Hierarchical Clustering and Data Visualizatio***n***

Unsupervised hierarchical clustering was performed in order to visualize and examine the expression of the Ψ-lncs in individual samples (Figure 2A). Average Linkage was used as a cluster distance metric, while Pearson Correlation was used as a point distance metric. To visualize the expression patterns of the most highly expressed Ψ-lncs, those with an average expression value of ≥ 10 RPKM in either tumor or non-malignant samples were included in the analysis.

#### Distribution of Deregulated Ψ-lncs Across Genome

Locations of Ψ-lncs and parent genes were compared to identify their genomic position (Supplementary Table S4). Circular plot visualization was performed using the R-package Circlize (Figure 3; Gu et al., 2014). LUAD-specific regions of significant recurrent somatic copy number alterations had been previously identified by TCGA, and were used in this study to determine if the deregulated Ψ-lncs overlapped with frequently altered regions (Zack et al., 2013). All genomic coordinates correspond to the NCBI GRCh37 reference human genome build.

### Clinical Features

#### Survival Analysis

A large public clinical database (Kaplan–Meier Plotter)^6^ comprised of 719 LUAD samples was used to determine the association between both protein-coding and non-coding gene expression with patient outcomes. Similar to the BCCA and TCGA cohorts, the patient samples in this 3rd dataset were mostly comprised of Stage I and Stage II tumors (Table 1). Of the 104 deregulated Ψ-lncs, 19 were represented in this database, while 70% of parent genes (72 out of 103) were present. Default settings were used and a log-rank (Mantel–Cox) test was applied to compare survival between groups of tumors with high and low expression of each gene tested, where *p* < 0.05 was considered statistically significant. The optimal expression cut-off was selected for each gene.

#### Association With Tumor Stage

The majority of tumors for BCCA and TCGA fell into the categories of Stage I and Stage II (Table 1). We compared expression of deregulated Ψ-lncs between Stage I tumors and tumors classified as Stage II and above using a Mann Whitney *U*-test (*p*-value ≤ 0.05).

## Author Contributions

GS was responsible for the project design. GS, KE, AS, VM, BM, MP, EM, and WL contributed to data acquisition, data analysis, interpretation of results, and manuscript preparation. WL was the principal investigator of this project. All authors have read, edited, and approved the final manuscript.

## Conflict of Interest Statement

The authors declare that the research was conducted in the absence of any commercial or financial relationships that could be construed as a potential conflict of interest.

## Footnotes

1 http://kmplot.com/analysis/

2 https://www.genenames.org/cgi-bin/statistics

3 http://www.ncbi.nlm.nih.gov/bioproject/516232

4 https://portal.gdc.cancer.gov/

5 https://www.random.org/

6 http://kmplot.com/analysis/

## Supplementary Material

FIGURE S1 Expression of lncRNA CTC-250I14.3 is significantly associated with Stage I disease in both the BCCA and TCGA LUAD cohorts (Mann Whitney *U*-test).

TABLE S1 Detailed information of all pseudogene-derived lncRNAs.

TABLE S2 Statistical analysis of the deregulated lncRNAs in the BCCA and TCGA cohorts (BHC-corrected *p*-values).

TABLE S3 Number of PUBMED entries for lncRNA cancer-association.

TABLE S4 Genomic locations of significant lncRNAs and their respective parent genes.

TABLE S5 Expression correlations between the lncRNAs and their resective parent genes in the BCCA and TCGA cohorts.

TABLE S6 Spearman’s correlation coefficients for global expression analysis of ψ-lnc-parent pairs.

TABLE S7 Parent gene information for pseudogenes contained in Retrolali5 and Yale60 databases.
