# Supplementary figures and images for: Aberrant Expression of Pseudogene-Derived lncRNAs as an Alternative Mechanism of Cancer Gene Regulation in Lung Adenocarcinoma
Source: Front Genet. 2019 Mar 6;10:138. doi: 10.3389/fgene.2019.00138 (PMC6414417; doi:10.3389/fgene.2019.00138)

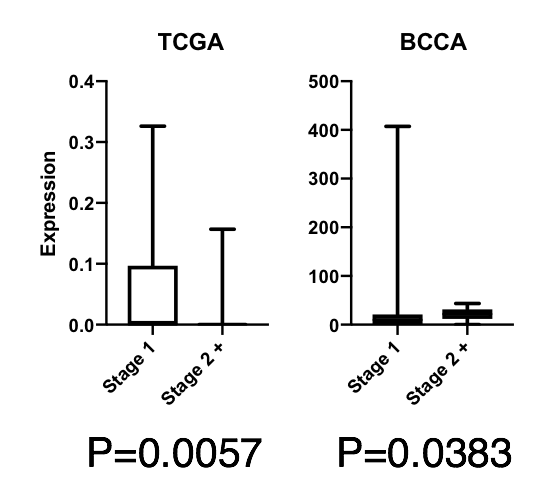

Supplement: FIGURE S1 — Expression of lncRNA CTC-250I14.3 is significantly associated with Stage I disease in both the BCCA and TCGA LUAD cohorts (Mann Whitney U-test). [file Image_1.PNG]
